# Dietary Nutrient Intake and Self-Reported Dermatitis/Eczema in U.S. Adults: A Survey-Weighted Cross-Sectional Analysis of National Health and Nutrition Examination Survey (NHANES) 1999-2004

**DOI:** 10.7759/cureus.117871

**Published:** 2026-10-13

**Authors:** Wed A Alawfi, Hadeel Aldhowayan, Rafif M Tahiri, Ghaday A Alrehaili, Hibah A Basehi, Nada A Algarni, Zainab W Kondarji, Raghad J Almuhaishi, Maryam J Alabdi

**Affiliations:** 1 Clinical Nutrition, College of Applied Medical Sciences, Taibah University, Medina, SAU; 2 College of Medicine, Jazan University, Jazan, SAU; 3 College of Medicine and Surgery, Taibah University, Medina, SAU; 4 Emergency Department, King Abdullah Medical Complex, Jeddah, SAU; 5 College of Medicine, Al-Rayan National Colleges, Medina, SAU; 6 College of Medicine, Imam Abdulrahman Bin Faisal University, Dammam, SAU; 7 College of Medicine, Vision College of Medicine, Riyadh, SAU

**Keywords:** dermatitis, diet, dietary intake, eczema, inflammatory skin disease

## Abstract

Introduction: Dermatitis and eczema are major chronic inflammatory skin conditions that may substantially affect quality of life. Data regarding the association between dietary intake and dermatitis/eczema remain limited and inconsistent. Thus, our aim is to characterize dietary differences between U.S. adults with and without self-reported dermatitis/eczema.

Methods: This is a survey-weighted cross-sectional analysis of pooled National Health and Nutrition Examination Survey (NHANES) 1999-2004 data among adults aged 20-59 years with valid Day 1 24-hour dietary recall data. Nutrient intake was compared by dermatitis/eczema status, and survey-weighted univariable and multivariable logistic regression models assessed associations between individual dietary nutrients and dermatitis/eczema. We used a random forest classifier with temporal validation (development on 1999-2002 data, testing on 2003-2004 data) and SHapley Additive exPlanations (SHAP)-based interpretation to evaluate the discriminative capacity of dietary variables.

Results: Among 8,702 participants, 1,076 (12.36%) reported dermatitis/eczema within the previous 12 months. Those affected tended to be older, more commonly non-Hispanic White, more educated, and more likely to be obese or former smokers. Overall dietary intake was similar across groups, with one exception: riboflavin was the only nutrient significantly linked to dermatitis/eczema, both in descriptive comparisons (2.29 ± 0.05 mg vs. 2.19 ± 0.03 mg, p=0.049) and in adjusted regression analysis (adjusted odds ratio (aOR) 1.075, 95% CI 1.001-1.154, p=0.046). A random forest model based solely on dietary variables showed only modest predictive ability (area under the receiver operating characteristic curve (AUROC) 0.531), performing worse than a model using clinical and demographic variables alone (AUROC 0.585). Combining both dietary and clinical/ demographic data did not improve predictive performance.

Conclusion: Except for a slight, borderline correlation with riboflavin, dietary nutrient consumption in U.S. adults exhibited little independent connection with self-reported dermatitis/eczema. For differentiating illness status, sociodemographic and clinical factors performed better than nutritional variables, indicating that diet has a minor impact compared with other factors.

## Introduction

Dermatitis, eczema, and related inflammatory skin conditions are chronic recurring disorders that weigh heavily on people and healthcare systems [1]. Atopic dermatitis affects not only children but also a significant number of adults in the United States. Studies indicate that its prevalence among adults ranges from 7.0% to 10.0% [2,3]. In adults, dermatitis and eczema cause more than skin problems; they also lead to poorer quality of life, trouble sleeping, and emotional strain [4].

The pathogenesis of dermatitis/eczema is characterized by a complex etiology, combining genetic predisposition, impaired skin barrier, and type 2 immune response caused by environmental triggers [5]. Oxidative stress and the accumulation of reactive oxygen species are widely considered to play a key role in keratinocyte damage and increase disease chronicity [6]. Based on the established connection between oxidative stress and cutaneous inflammation, recent investigations have evaluated the potential of antioxidants to modulate both the incidence and the clinical severity associated with dermatitis and eczema [7].

Whilst a plausible biological rationale exists to support a potential association between dietary nutrient intake and the risk or severity of dermatitis/eczema, evidence among adults remains limited, and findings across the existing literature have been inconsistent. Several earlier studies were conducted in non-United States populations, while others examined serum biochemical measures rather than dietary intake itself. These methodological limitations indicate that the relationship between dietary intake and dermatitis/eczema in adults remains insufficiently understood [8-10].

Using data from the National Health and Nutrition Examination Survey (NHANES), this study aimed to compare the sociodemographic and clinical characteristics of U.S. individuals with and without dermatitis/eczema and to assess dietary intake between the two groups using survey-weighted univariable and multivariable-adjusted logistic regression, applying national survey weights. As a secondary objective, a random forest classifier with temporal validation and SHapley Additive exPlanations (SHAP)-based interpretation was used to investigate whether a multidimensional dietary profile could distinguish individuals with dermatitis/eczema.

## Materials and methods

Data source and study population

This study utilized data from three consecutive NHANES cycles: 1999-2000, 2001-2002, and 2003-2004. NHANES is a nationally representative, cross-sectional survey that utilizes a complex, multistage probability sampling design to evaluate health-related and nutritional status of non-institutionalized individuals in the United States [11]. All analyses in this study have accounted for this complex design, including the appropriate dietary sampling weights, masked variance strata, and primary sampling units. Dietary recall weights have been used for the purpose of this study in preference to interview or examination weights because the primary dietary variable used was derived from the Day 1 24-hour dietary recall. We pooled the NHANES cycles through which the outcome variable was present and consistent.

Since not all nutrients are available throughout the 1999-2004 period, pooled six-year dietary weights were used for nutrients available throughout the six-year period by assigning two-thirds of the 1999-2002 four-year dietary weight (WTDR4YR) to participants from the 1999-2002 cycles and one-third of the 2003-2004 Day 1 dietary weight (WTDRD1) to participants from the 2003-2004 cycle. For nutrients which were only available in the 2001-2004 survey, a corresponding four-year dietary weight, calculated as one-half of the two-year Day 1 dietary weight for each included cycle were used. Appropriate survey strata and primary sampling unit identifiers were treated as cycle-specific when cycles were combined.

The population included in the analysis was exclusive to adults from 20 to 59 years of age to ensure consistency in age-eligibility across the survey cycles. Furthermore, a reliable Day 1 dietary recall and a positive, applicable dietary sampling weight were required for participants to be included in our analysis.

Primary outcome

Self-reported presence of dermatitis, eczema, or another red or inflamed skin rash during the preceding 12 months was the primary dependent variable, analyzed as a binary variable (yes vs. no). Participants who refused to answer, or those with unknown or missing responses, were excluded from outcome-specific analyses.

Dietary exposure assessment

Nutritional intake was assessed using the Day 1 24-hour dietary recall variable. Kilocalories per day were used to analyze total energy intake. Non-energy nutrients were expressed as nutrient intake per 1,000 kcal to reduce confounding by total caloric intake. In regard to regression analyses, each dietary exposure was standardized using its survey weighted standard deviation; therefore, odds ratios represent the change in the odds of dermatitis/eczema associated with a 1-standard-deviation increase in the corresponding dietary exposure.

Covariates

Several sociodemographic and clinical covariates were included in our analysis, such as age, sex, race/ethnicity, educational level, body mass index (BMI) categories, smoking status, alcohol use, and diabetes mellitus. Total energy intake was also included as a covariate for all non-energy nutrient models.

Statistical analysis

Participant characteristics were summarized according to dermatitis/eczema status. Categorical variables were presented as unweighted frequencies with column percentages, whereas continuous variables were summarized using survey-weighted means and standard errors (SEs). Between-group comparisons were performed using survey-adjusted tests that incorporated sampling weights, strata, and clustering. Normality of continuous variables was evaluated using the Shapiro-Wilk test. Although dietary variables demonstrated statistically significant departures from normality, the large sample size and the population-estimation objective of the complex survey analysis supported presentation of survey-weighted means and SEs.

Initially, separate survey-weighted univariable binary logistic regression models were fitted for each dietary nutrient, with dermatitis/eczema status as the dependent variable. Results were reported as odds ratios (ORs), 95% confidence intervals (CIs), and P values. Each nutrient was entered into a separate model to avoid instability and multicollinearity resulting from the substantial correlation among dietary variables.

Multivariable survey-weighted binary logistic regression models were subsequently used to evaluate the independent association between individual dietary nutrients and dermatitis/eczema. Each nutrient was modeled separately while adjusting for prespecified demographic and clinical covariates, including age, sex, race/ethnicity, educational attainment, body mass index category, smoking status, alcohol use, diabetes mellitus, survey cycle, and total energy intake for all non-energy nutrient models. Adjusted odds ratios (aORs) with 95% CIs were reported. A two-sided P value <0.05 was considered nominally statistically significant, whereas q<0.05 was considered statistically significant after correction for multiple comparisons. Q-values were obtained using the Benjamini-Hochberg false discovery rate procedure applied across all 29 nutrient models, separately for the univariable and multivariable analyses.

As a secondary exploratory analysis, a machine-learning approach was used to assess whether multidimensional dietary patterns could discriminate participants with versus without dermatitis/eczema. A Random Forest classifier was developed using dietary variables that were consistently available across the 1999-2004 cycles. To provide temporal rather than random internal validation, data from NHANES 1999-2002 were used for model development and data from NHANES 2003-2004 were reserved as an independent temporal test set. Dietary sampling weights were incorporated as sample weights during model fitting and performance evaluation.

Random Forest performance in the temporal test set was evaluated using the area under the receiver operating characteristic curve (AUROC), area under the precision-recall curve (AUPRC), sensitivity, specificity, precision, F1 score, and Brier score. Because dermatitis/eczema represented a minority of the analytic sample, AUPRC was considered alongside AUROC when evaluating discrimination. The classification threshold used for sensitivity and specificity estimation was selected within the development data rather than the temporal test set.

Model interpretability was assessed using SHAP. SHAP values were used to quantify the contribution of individual dietary variables to Random Forest predictions. Positive SHAP values indicated that a given dietary exposure shifted the model prediction toward dermatitis/eczema, whereas negative values indicated a shift toward the absence of dermatitis/eczema.

Statistical analyses were performed using Stata 15.1 (StataCorp LLC, College Station, TX, USA), and Python 3.8 (Python Software Foundation, Fredericksburg, VA, USA) and Matplotlib were utilized for the purpose of data visualization.

## Results

Sample characteristics

Sample characteristics are shown in Table 1. In our analysis, we included 8,702 U.S. civilians, with ages ranging from 20 to 59 years. A total of 1,076 (12.36%) reported dermatitis, eczema, or another red, inflamed skin rash in the past 12 months, while 7,626 (87.64%) did not. Individuals who reported dermatitis/eczema were less likely to be male compared to those without dermatitis/eczema (43.4% vs. 46.8%, p=0.047) and were older than non-dermatitis/eczema individuals on average (39.27 ± 10.94 years vs. 37.73 ± 11.20 years, p=0.002). Furthermore, a distinctive pattern was noticed across race/ethnicity, educational level, BMI, and smoking status. Non-Hispanic White individuals constituted a larger percentage of those with dermatitis/eczema compared to those without (59.9% vs. 44.8%), whereas Mexican American adults constituted a smaller percentage of the dermatitis/eczema group (14.8% vs. 24.6%; overall p<0.001 for ethnicity). Moreover, higher educational level was noted among those with dermatitis/eczema, with around 62.8% having education greater than high school compared with 47.1% of those without dermatitis/eczema (p<0.001). In regard to BMI, obesity was significantly more prevalent among individuals reporting dermatitis/eczema (32.0% vs. 28.2%), while the overweight BMI category was less prevalent (29.6% vs. 33.4%; overall p=0.03). Also, among the dermatitis/eczema group, never-smoking status had a smaller proportion compared to those without dermatitis/eczema (49.0% vs. 54.7%), while former smoking had a higher prevalence rate (23.2% vs. 18.6%, overall p=0.04). As for alcohol consumption and diabetes, no statistical difference was observed.

**Table 1:** Characteristics of U.S. adults according to self-reported dermatitis/eczema status, NHANES 1999-2004 Values are unweighted n (column %) unless otherwise specified. Percentages are calculated using the total unweighted number of participants in each column as the denominator (Overall n = 8,702; No dermatitis n = 7,626; Dermatitis n = 1,076). Age is shown as a survey-weighted mean. P values account for the NHANES complex survey design, including dietary recall weights, strata, and primary sampling units; the age comparison used survey-weighted linear regression, and categorical variables used survey-adjusted global Wald F tests. NHANES: National Health and Nutrition Examination Survey, GED: general educational development, BMI: body mass index

	Total	No dermatitis	Dermatitis	p-value
Number (%)	8702	7626 (87.64)	1076 (12.36)	
Gender (male, n [%])	4,036 (46.4%)	3,569 (46.8%)	467 (43.4%)	0.047
Age (years ± [SD])	37.92 ± 11.18	37.73 ± 11.20	39.27 ± 10.94	0.002
Ethnicity				
Mexican American (Yes, n [%])	2,037 (23.4%)	1,878 (24.6%)	159 (14.8%)	<0.001
Other Hispanic (Yes, n [%])	453 (5.2%)	405 (5.3%)	48 (4.5%)	
Non-Hispanic White (Yes, n [%])	4,062 (46.7%)	3,417 (44.8%)	645 (59.9%)	
Non-Hispanic Black (Yes, n [%])	1,795 (20.6%)	1,626 (21.3%)	169 (15.7%)	
Other/multiracial (Yes, n [%])	355 (4.1%)	300 (3.9%)	55 (5.1%)	
Education				
Less than high school	2,341 (26.9%)	2,177 (28.5%)	164 (15.2%)	<0.001
High school/GED (Yes, n [%])	2,083 (23.9%)	1,848 (24.2%)	235 (21.8%)	
>High school (Yes, n [%])	4,270 (49.1%)	3,594 (47.1%)	676 (62.8%)	
BMI				
Underweight (Yes, n [%])	134 (1.5%)	117 (1.5%)	17 (1.6%)	0.03
Healthy weight (Yes, n [%])	2,967 (34.1%)	2,583 (33.9%)	384 (35.7%)	
Overweight (Yes, n [%])	2,868 (33.0%)	2,549 (33.4%)	319 (29.6%)	
Obese (Yes, n [%])	2,498 (28.7%)	2,154 (28.2%)	344 (32.0%)	
Smoking status				
Never (Yes, n [%])	4,699 (54.0%)	4,172 (54.7%)	527 (49.0%)	0.04
Former (Yes, n [%])	1,665 (19.1%)	1,415 (18.6%)	250 (23.2%)	
Current (Yes, n [%])	2,329 (26.8%)	2,030 (26.6%)	299 (27.8%)	
≥12 drinks in any one year (Yes, n [%])	5,851 (67.2%)	5,078 (66.6%)	773 (71.8%)	0.1
Diabetes (Yes, n [%])	452 (5.2%)	388 (5.1%)	64 (5.9%)	0.06

Dietary nutrient intake by dermatitis/eczema status

Survey-weighted mean dietary nutrient intake was comparable between both the dermatitis/eczema group and the non-dermatitis/eczema group (Table 2). Riboflavin intake stands as the only significant nutrient, which was significantly higher among individuals with dermatitis/eczema (2.29 ± 0.05 mg vs. 2.19 ± 0.03 mg, p=0.049). Mean total energy intake did not show a statistically significant difference between groups (2,338.56 ± 38.80 kcal vs. 2,322.44 ± 17.33 kcal, p=0.693). Furthermore, no statistically significant difference was observed in micronutrient intake, including protein, carbohydrate, total sugars, dietary fiber, total fat, or fat subtypes, between individuals with dermatitis/eczema and those without (all p>0.05). Likewise, no significant difference was observed for cholesterol, vitamins (vitamin E, retinol, vitamin A, alpha- and beta carotene, thiamin, niacin, vitamin B6, folate, folic acid, vitamin B12, vitamin C, or vitamin K), and minerals (calcium, phosphorus, magnesium, iron, or zinc; all p>0.05).

**Table 2:** Survey-weighted dietary nutrient intake according to dermatitis/eczema status Values are survey-weighted mean ± standard error (SE). Standard errors were estimated using Taylor-series linearization accounting for the NHANES dietary recall weights, strata, and primary sampling units. P values compare participants with and without dermatitis/eczema using survey-adjusted tests. Nutrients available across 1999-2004 use the six-year Day-1 dietary weight; nutrients available only in 2001–2004 use the corresponding four-year Day-1 dietary weight. RAE: retinol activity equivalents

	No dermatitis	Dermatitis	p-value
Number (%)	7626 (87.64)	1076 (12.36)	
Energy (kcal) (mean ± [SE])	2322.44 ± 17.33	2338.56 ± 38.80	0.693
Protein (g) (mean ± [SE])	85.79 ± 0.71	87.06 ± 1.62	0.459
Carbohydrate (g) (mean ± [SE])	287.18 ± 2.43	288.74 ± 6.00	0.790
Total sugars (g) (mean ± [SE])	138.74 ± 2.17	146.95 ± 4.69	0.116
Dietary fiber (g) (mean ± [SE])	15.82 ± 0.26	16.45 ± 0.60	0.291
Total fat (g) (mean ± [SE])	86.44 ± 0.72	88.48 ± 1.68	0.259
Saturated fat (g) (mean ± [SE])	28.59 ± 0.28	29.33 ± 0.70	0.329
Monounsaturated fat (g) (mean ± [SE])	32.44 ± 0.28	33.11 ± 0.66	0.350
Polyunsaturated fat (g) (mean ± [SE])	17.71 ± 0.18	18.28 ± 0.43	0.208
Cholesterol (mg) (mean ± [SE])	298.71 ± 4.11	286.93 ± 9.31	0.276
Vitamin E/alpha-tocopherol (mg) (mean ± [SE])	7.90 ± 0.12	8.31 ± 0.30	0.210
Retinol (mcg) (mean ± [SE])	421.99 ± 15.72	461.17 ± 26.31	0.147
Vitamin A (mcg RAE) (mean ± [SE])	719.09 ± 18.34	718.38 ± 26.21	0.978
Alpha-carotene (mcg) (mean ± [SE])	372.93 ± 19.76	403.37 ± 58.57	0.636
Beta-carotene (mcg) (mean ± [SE])	1864.48 ± 69.05	2107.07 ± 218.16	0.292
Thiamin (mg) (mean ± [SE])	1.69 ± 0.02	1.71 ± 0.04	0.485
Riboflavin (mg) (mean ± [SE])	2.19 ± 0.03	2.29 ± 0.05	0.049
Niacin (mg) (mean ± [SE])	24.41 ± 0.26	24.68 ± 0.56	0.659
Vitamin B6 (mg) (mean ± [SE])	1.91 ± 0.03	1.97 ± 0.05	0.216
Total folate (mcg) (mean ± [SE])	408.11 ± 6.43	425.68 ± 14.49	0.239
Folic acid (mcg) (mean ± [SE])	202.74 ± 4.71	205.34 ± 8.94	0.789
Vitamin B12 (mcg) (mean ± [SE])	5.26 ± 0.14	5.74 ± 0.31	0.123
Vitamin C (mg) (mean ± [SE])	92.85 ± 2.82	99.40 ± 6.62	0.272
Vitamin K (mcg) (mean ± [SE])	88.67 ± 3.30	96.29 ± 7.28	0.337
Calcium (mg) (mean ± [SE])	892.97 ± 12.49	942.55 ± 27.34	0.086
Phosphorus (mg) (mean ± [SE])	1380.12 ± 12.35	1429.32 ± 24.81	0.065
Magnesium (mg) (mean ± [SE])	288.22 ± 3.51	301.51 ± 6.59	0.059
Iron (mg) (mean ± [SE])	15.91 ± 0.19	16.25 ± 0.36	0.339
Zinc (mg) (mean ± [SE])	12.41 ± 0.14	12.58 ± 0.30	0.559

Association between dietary nutrient intake and dermatitis/eczema

When assessing the association between dietary nutrients and dermatitis/eczema through logistic regression analysis, riboflavin intake stands as the only dietary nutrient with a significant association with dermatitis/eczema, with each 1-standard deviation increase in intake being associated with 7.5% higher odds of dermatitis/eczema (OR 1.075, 95% CI 1.001-1.154, p=0.046) (Table 3, Figure 1). No other nutrient has achieved statistical significance, although multiple nutrients have approached the nominal threshold and showed associations, such as magnesium (OR 1.086, 95% CI 0.999-1.180, p=0.053), phosphorus (OR 1.070, 95% CI 0.996-1.149, p=0.063), calcium (OR 1.076, 95% CI 0.991-1.168, p=0.078), and total sugars (OR 1.088, 95% CI 0.983-1.203, p=0.099). In contrast, odds ratios of all other nutrients, including total energy intake, micronutrients, fat subtypes, cholesterol, vitamins, and minerals, have narrowly ranged around the null value and showed no statistical significance (Table 3).

**Table 3:** Univariate survey-weighted logistic regression analysis of dietary nutrient intake associated with dermatitis/eczema The binary outcome was past-year dermatitis/eczema/red inflamed skin rash (1 = yes; 0 = no). Each dietary nutrient was entered separately in a survey-weighted binary logistic regression model. ORs are reported per 1 survey-weighted standard-deviation increase in nutrient intake. Models accounted for NHANES dietary sampling weights, strata, and primary sampling units. Total sugars, retinol, alpha-carotene, beta-carotene, folic acid, and vitamin K were analyzed using 2001-2004 dietary weights because these variables were not available across the full 1999-2004 period. No covariates were included in these univariate models. OR: odds ratio, CI: confidence interval, RAE: retinol activity equivalents, NHANES: National Health and Nutrition Examination Survey

Dietary nutrient	OR	95% CI	P value
Energy (kcal)	1.015	0.941–1.095	0.691
Protein (g)	1.029	0.954–1.109	0.452
Carbohydrate (g)	1.011	0.932–1.097	0.789
Total sugars (g)	1.088	0.983–1.203	0.099
Dietary fiber (g)	1.060	0.954–1.178	0.271
Total fat (g)	1.042	0.970–1.119	0.252
Saturated fat (g)	1.042	0.960–1.131	0.321
Monounsaturated fat (g)	1.034	0.964–1.110	0.342
Polyunsaturated fat (g)	1.048	0.976–1.125	0.195
Cholesterol (mg)	0.951	0.864–1.046	0.291
Vitamin E/alpha-tocopherol (mg)	1.059	0.973–1.152	0.182
Retinol (mcg)	1.043	0.981–1.108	0.172
Vitamin A (mcg RAE)	0.999	0.951–1.050	0.978
Alpha-carotene (mcg)	1.022	0.937–1.115	0.608
Beta-carotene (mcg)	1.058	0.961–1.165	0.241
Thiamin (mg)	1.026	0.954–1.105	0.478
Riboflavin (mg)	1.075	1.001–1.154	0.046
Niacin (mg)	1.019	0.936–1.110	0.655
Vitamin B6 (mg)	1.050	0.973–1.134	0.202
Total folate (mcg)	1.066	0.963–1.179	0.213
Folic acid (mcg)	1.013	0.923–1.111	0.786
Vitamin B12 (mcg)	1.042	0.989–1.097	0.120
Vitamin C (mg)	1.057	0.964–1.158	0.229
Vitamin K (mcg)	1.047	0.957–1.145	0.310
Calcium (mg)	1.076	0.991–1.168	0.078
Phosphorus (mg)	1.070	0.996–1.149	0.063
Magnesium (mg)	1.086	0.999–1.180	0.053
Iron (mg)	1.035	0.965–1.110	0.326
Zinc (mg)	1.020	0.957–1.086	0.543

**Figure 1:**
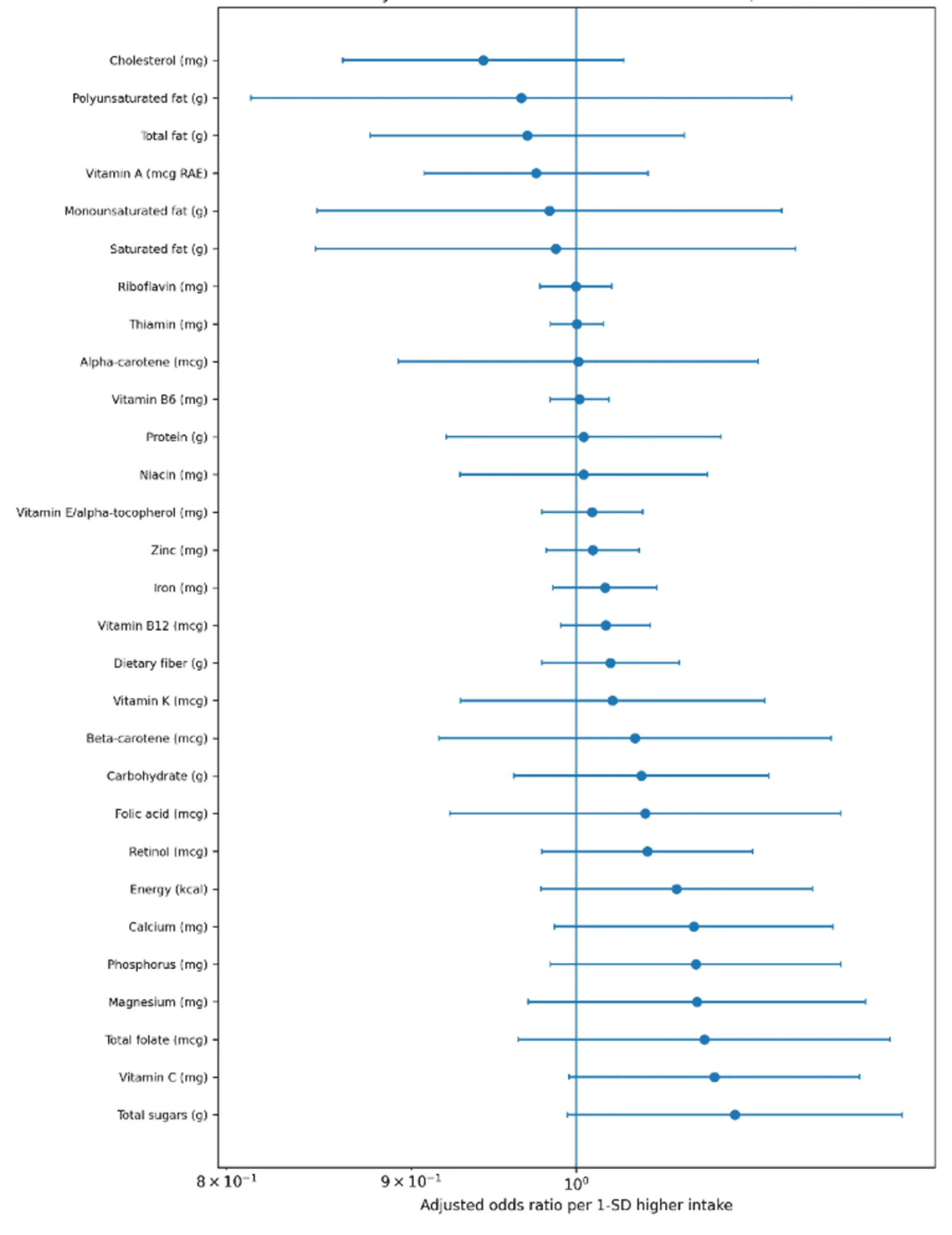
Multivariate analysis of the association between dietary nutrient intake and self-reported dermatitis/eczema among U.S. adults, NHANES 1999-2004 Forest plot shows the association between individual dietary nutrient intakes and the odds of self-reported dermatitis/eczema. Each nutrient was analyzed separately using survey-weighted binary logistic regression, with dermatitis/eczema as the binary outcome. Odds ratios (ORs) and 95% confidence intervals (CIs) are presented per 1 standard-deviation increase in nutrient intake. Analyses incorporated the appropriate NHANES dietary sampling weights, strata, and primary sampling units. Nutrients available only during 2001-2004 were analyzed using the corresponding four-year dietary weights, whereas nutrients available across 1999-2004 were analyzed using the six-year dietary weights. All models were adjusted for age, sex, race/ethnicity, educational attainment, body mass index category, smoking status, alcohol use, diabetes mellitus, and survey cycle; models for non-energy nutrients were additionally adjusted for total energy intake. The vertical reference line at OR = 1.0 indicates no association. RAE: retinol activity equivalents, NHANES: National Health and Nutrition Examination Survey

Machine-learning prediction of dermatitis/eczema from dietary intake

In the NHANES 2003-2004 temporal test sample, an overall modest discriminative performance was achieved by the machine-learning models built from dietary nutrient variables (Table 4). The highest AUROC was reached by the random forest classifier (0.531) and was carried forward for subsequent predictor-set comparisons, with corresponding performance parameters: AUPRC of 0.149, sensitivity and specificity of 0.447 and 0.600, respectively. AUROCs of other models ranged from 0.502 (gradient boosting) to 0.523 (elastic net), achieving lower results. Interestingly, the elastic net model achieved higher sensitivity of 0.843 but a markedly lower specificity of 0.179, reflecting a potential bias towards categorizing individuals as having dermatitis/eczema.

**Table 4:** Temporal validation performance of machine-learning models for predicting dermatitis/eczema from dietary nutrient intake Performance was evaluated in the independent 2003-2004 temporal test sample using NHANES dietary sampling weights. Sensitivity, specificity, precision, and F1 score were calculated using a threshold selected in the internal development-validation sample by the weighted Youden index. The Random Forest had the highest test AUROC and was carried forward for predictor-set comparison. AUROC: area under the receiver operating characteristic curve, AUPRC: area under the precision-recall curve, NHANES: National Health and Nutrition Examination Survey.

Model	AUROC	AUPRC	Sensitivity	Specificity
Random Forest	0.531	0.149	0.447	0.6
Elastic Net	0.523	0.157	0.843	0.179
Logistic regression	0.519	0.154	0.414	0.611
XGBoost	0.508	0.148	0.771	0.256
Gradient Boosting	0.502	0.144	0.835	0.191

Upon predictor sets comparison with the use of the random forest algorithm (Table 5), the lowest discrimination value was reached by the dietary-nutrients-only model (AUROC 0.531, AUPRC 0.149). Modestly higher discrimination was achieved by the model restricted to clinical/demographic predictors (age, sex, race/ethnicity, education, body mass index, smoking status, alcohol history, and diabetes) (AUROC 0.585, AUPRC 0.204; Δ AUROC +0.054 versus the dietary-only model). When both models are combined (dietary-plus-clinical/demographic model), no improvement is seen over the clinical/demographic-only model (AUROC 0.582, AUPRC 0.185). This indicates that dietary nutrient intake did not meaningfully augment discrimination beyond demographic and clinical factors alone. All three predictor sets achieved similar Brier scores (0.124-0.125), which is consistent with comparably modest overall calibration and predictive performance.

**Table 5:** Comparison of dietary, clinical/demographic, and combined predictor sets using random forest The clinical/demographic predictor set included age, sex, race/ethnicity, education, body mass index, smoking status, alcohol history, and diabetes. Delta AUROC is calculated relative to the dietary-only Random Forest. The combined model did not improve discrimination beyond the clinical/demographic-only model. AUROC: area under the receiver operating characteristic curve, AUPRC: area under the precision-recall curve

Predictor set	AUROC	AUPRC	Delta AUROC vs dietary	Brier score
Dietary nutrients only	0.531	0.149	0	0.125
Clinical/demographic only	0.585	0.204	0.054	0.124
Dietary + clinical/demographic	0.582	0.185	0.05	0.124

Utilizing SHAP analysis of dietary fat variables within the random forest model (Figure 2), individual contribution of fat subtype intake (saturated fat, total fat, and polyunsaturated fat) was illustrated in relation to model predictions, with non-uniform, divergent shifts in the predicted probability of dermatitis/eczema occurrence associated with higher and lower nutrient intake values. This, therefore, contradicts the belief of a simple monotonic association rather than a more complex, non-linear relationship captured by the random forest algorithm.

**Figure 2:**
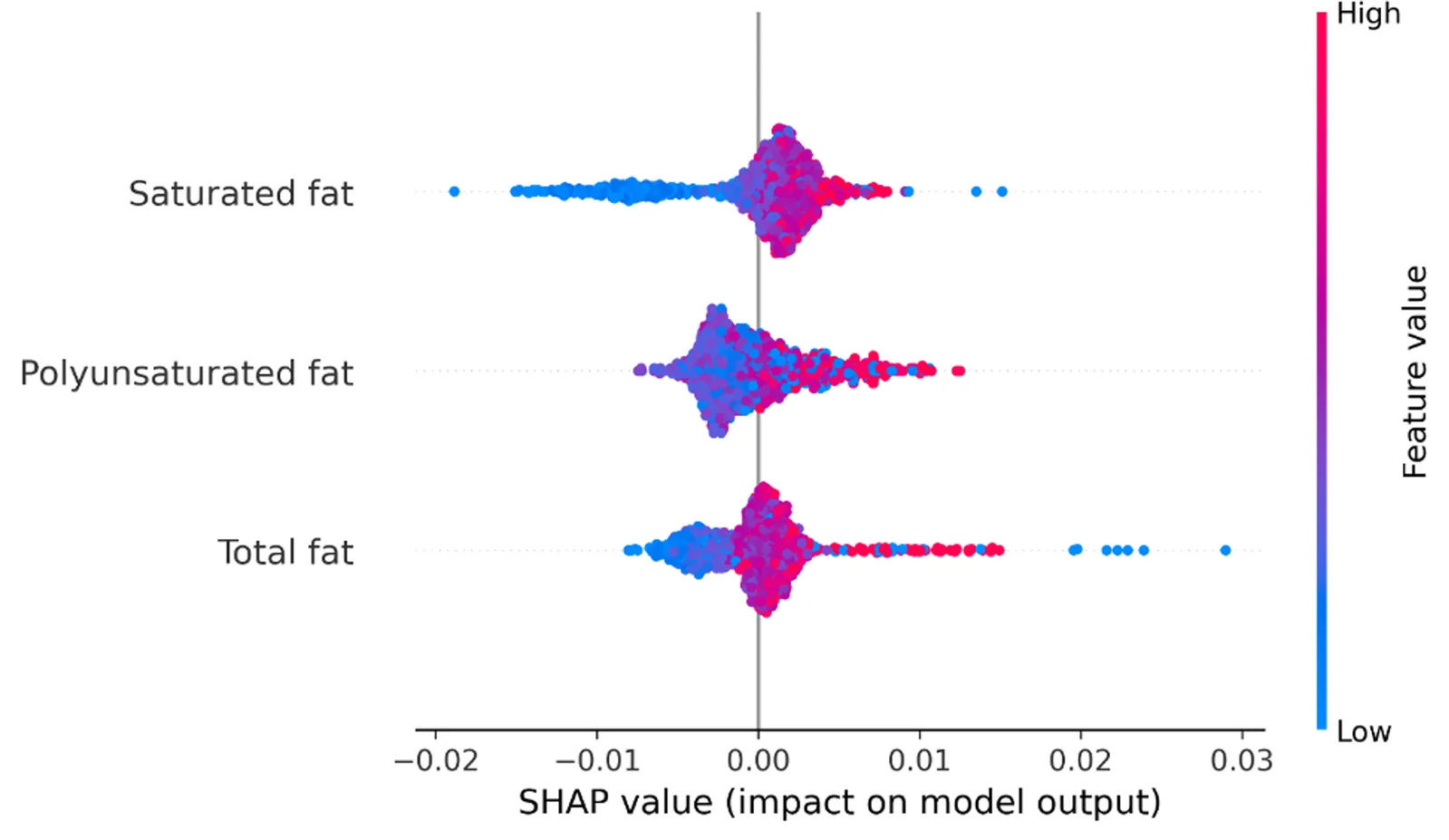
SHAP summary plot of dietary fat variables in the random forest model for dermatitis/eczema SHAP (SHapley Additive exPlanations) summary plot illustrating the contribution of saturated fat, total fat, and polyunsaturated fat intake to predictions generated by the Random Forest model for self-reported dermatitis/eczema. Each point represents an individual participant. The horizontal axis shows the SHAP value, with positive values indicating that the corresponding dietary fat intake shifted the model prediction toward dermatitis/eczema and negative values indicating a shift toward no dermatitis/eczema. Point color represents the relative magnitude of nutrient intake, with red indicating higher values and blue indicating lower values. SHAP values reflect the contribution of each predictor to the model prediction and should not be interpreted as odds ratios, measures of statistical significance, or causal effects.

## Discussion

In our nationally representative, cross-sectional analysis of NHANES 1999-2004, self-reported occurrence of dermatitis/eczema was shown to be associated with modest sociodemographic differences, such as older age, non-Hispanic White race, higher educational level, obesity, and a lower percentage of never-smokers. This pattern fairly aligns with previous population-based studies on the U.S. population, which have demonstrated sociodemographic differences related to eczema in terms of age, race/ethnicity, and socioeconomic factors [2,12]. On the contrary, dietary nutrient intake showed a large similarity between both groups, with riboflavin intake being the only significant nutrient associated with dermatitis/eczema in both descriptive and regression analyses.

Our finding that riboflavin intake was associated with modestly higher, rather than lower, odds of dermatitis/eczema (OR 1.075, 95% CI 1.001-1.154) is noteworthy and warrants cautious interpretation. Our finding is directionally inconsistent with previously published evidence in the literature among young Chinese adult individuals, in which lower odds of dermatitis/eczema were linked to higher intake of vitamin B2 (along with vitamins E, K, C, and D) [13]. This conflicting finding can have several explanations. First, riboflavin has been shown to have photosensitizing features, as in the presence of ultraviolet A (UVA) radiation, riboflavin contributes to the generation of hydrogen peroxide and other reactive oxygen species in the skin, a mechanism that has been implicated in UVA-induced cellular damage of dermal fibroblasts [14]. Thus, biological plausibility supports the theory that in a group of individuals, high riboflavin intake can contribute to cutaneous oxidative stress under sunlight exposure, providing contradictory evidence to the widely reported evidence supporting riboflavin’s antioxidant and anti-inflammatory roles [7]. Second, reverse causation or behavioral changes after the occurrence of dermatitis/eczema, such as dietary modification, supplementation, or differential recall, cannot be ruled out, especially given that dietary intake was evaluated using a one-day 24-hour dietary intake recall. The borderline riboflavin finding may be a result of testing many nutrients rather than a true association; thus, results should be interpreted with caution. 

For all other nutrients, the absence of significance in association contrasts with studies reported in the literature linking certain dietary patterns to atopic dermatitis, such as the consumption of processed or instant foods [8,9]. This discrepancy might result from differences in study populations (young, Asian cohorts vs. a broader U.S. adult population), outcome definition (clinically confirmed vs. self-reported dermatitis/eczema/rash), or dietary method assessment (validated questionnaires vs. single-day 24-hour dietary intake recall). Furthermore, it is also a possibility that true effects of dietary intake on dermatitis/eczema are minute in magnitude and are difficult to detect using single-day recall data, which could be subject to considerable day-to-day variability and might not reflect long-term intake.

Machine-learning models were used to further assess the largely null findings, in which only marginal discrimination between individuals with and without dermatitis/eczema has been achieved in an independent temporal test sample using a random forest model built exclusively from dietary intake variables (AUROC 0.531). Notably, a model based only on demographic and clinical covariates outperformed the dietary-only model and was not improved further after combining dietary intake with clinical/demographic factors. Taken together, our observation suggests that within our framework, single-day dietary intake holds only a limited independent capacity for discrimination of dermatitis/eczema relative to sociodemographic and clinical characteristics. This pattern is consistent with the substantial contribution of genetic, immunologic, and skin-barrier factors to disease pathogenesis reported in the literature [5,6]. Our SHAP-based analysis of fat subtype intake has revealed a complex, non-linear relationship between fat intake and model predictions, suggesting that fat-dermatitis relationships in the population are largely non-uniform or are effect-modified rather than a relationship that can be adequately captured by a simple linear odds ratio. This hypothesis aligns with the recently described non-linear relationship between vitamin E levels in serum and eczema in U.S. adults [10].

Several limitations are present in this study. First, the nature of cross-sectionally designed studies precludes temporal or causal inferences regarding the relationship between dietary intake and the occurrence of dermatitis/eczema. Second, the outcome of interest in our study was based on a self-report of dermatitis, eczema, or red/inflamed rash rather than a clinically established diagnosis, which might result in misclassification and inability to distinguish between the subtypes of dermatitis (e.g. atopic dermatitis, contact dermatitis, or seborrheic dermatitis). Third, dietary intake was based on a single-day 24-hour dietary intake recall, which can reflect only casual rather than habitual, long-term dietary intake, subjecting it to recall bias and considerable intra-individual variability. Fourth, residual confounding factors that were not gathered in the NHANES survey, such as genetic predisposition, environmental exposures, and skincare practices, could not be accounted for. Finally, the NHANES 1999-2004 cycles predate significant shifts in the food supply and dietary patterns observed recently, which can limit the generalizability of our observations to contemporary dietary habits.

Notwithstanding the limitations described, our study benefits from a large, nationally representative, survey-weighted sample with appropriate handling of the NHANES complex design, a comprehensive dietary panel, and a dual analytical approach utilizing traditional regression with machine-learning-based patterns and SHAP interpretability, along with a temporally validated predictive model. Further research prioritizing prospective designs with clinically confirmed diagnosis of dermatitis and validated dietary intake questionnaires should be conducted to confirm the relationship between nutrient intake and dermatitis/eczema. 

## Conclusions

In this nationally representative study, dietary nutrient intake showed limited independent association with self-reported dermatitis/eczema, except for a slight, borderline significant association with riboflavin intake. Machine-learning models demonstrated limited discrimination for dietary nutrient intake alone and did not outperform demographic and clinical factors. This suggests that looking at single-day food intake alone may not be very helpful for identifying U.S. adults with and without dermatitis/eczema compared with considering other non-food-related factors. Further studies with clinically confirmed dermatitis/eczema and more detailed assessment of usual dietary intake are needed.
